# The mitochondrial protease HtrA2 restricts the NLRP3 and AIM2 inflammasomes

**DOI:** 10.1038/s41598-018-26603-1

**Published:** 2018-05-31

**Authors:** Ian Gaël Rodrigue-Gervais, Karine Doiron, Claudia Champagne, Lindsey Mayes, Gabriel André Leiva-Torres, Paulin Vanié, Todd Douglas, Silvia M. Vidal, Emad S. Alnemri, Maya Saleh

**Affiliations:** 1 Department of Medicine, McGill University, Montréal, Québec, H3G 0B1 Canada; 2 INRS-Institut Armand Frappier, Laval, Québec, H7V 1B7 Canada; 3 Department of Biochemistry and Molecular Biology, Kimmel Cancer Center, Thomas Jefferson University, Philadelphia, Pennsylvania, PA 19107 USA; 4 Department of Human Genetics, McGill University, Montréal, Québec, H3A 1B1 Canada; 5 Department of Microbiology and Immunology, McGill University, Montréal, Québec, H3A 2B4 Canada; 6 Department of Biochemistry, McGill University, Montréal, Québec, H3G 1Y6 Canada

## Abstract

Activation of the inflammasome pathway is crucial for effective intracellular host defense. The mitochondrial network plays an important role in inflammasome regulation but the mechanisms linking mitochondrial homeostasis to attenuation of inflammasome activation are not fully understood. Here, we report that the Parkinson’s disease-associated mitochondrial serine protease HtrA2 restricts the activation of ASC-dependent NLRP3 and AIM2 inflammasomes, in a protease activity-dependent manner. Consistently, disruption of the protease activity of HtrA2 results in exacerbated NLRP3 and AIM2 inflammasome responses in macrophages *ex vivo* and systemically *in vivo*. Mechanistically, we show that the HtrA2 protease activity regulates autophagy and controls the magnitude and duration of inflammasome signaling by preventing prolonged accumulation of the inflammasome adaptor ASC. Our findings identify HtrA2 as a non-redundant mitochondrial quality control effector that keeps NLRP3 and AIM2 inflammasomes in check.

## Introduction

The inflammasomes are central innate immunity effectors that defend the host from infections and a variety of intrinsic or environmental insults^1^. These multi-protein complexes are scaffolded by pattern recognition receptors, which upon danger sensing promote the recruitment and activation of caspase-1. The protein ASC (apoptosis-associated speck-like protein containing a caspase-recruitment domain) acts as an essential adaptor that connects caspase-1 to the sensor in some but not all inflammasomes^1^. The NOD-like receptor, NLRP3, and the interferon-inducible protein AIM2 represent two of the most studied ASC-dependent inflammasome receptors. AIM2 binds to cytosolic DNA, including that of viral origin^2,3^, and NLRP3 is activated by several danger signals^1^, including viral nucleic acids through the endoribonuclease activity of RNase L^4^. In parallel to proteolytic maturation of IL-1 family cytokines, caspase-1 cleaves gasdermin D into an amino-terminal pore-forming domain that inserts into the cell membrane resulting in IL-1β release and a rapid lytic cell death known as pyroptosis^5–7^. However, IL-1β release can occur without pyroptosis in some contexts for e.g. following alternative activation of the inflammasome^8^. In viral infection, pyroptosis can have a complex effect on infection dynamics and host survival. It can eliminate the viral replication niche^9^, providing fuel for pro-inflammatory and reparative processes necessary in the resolution phase^10^, it can also cause host tissue damage, increasing viral illness severity^11–15^.

The mitochondrial network, which is central for energy production, metabolism and innate immune signaling, is highly susceptible to physiological and environmental stressors, including viral infections^16^. Its dynamics is maintained by fission and fusion, in tandem with mitophagy, a mitochondria-selective autophagy, and these processes have been linked to inflammasome regulation^17,18^. Indeed, autophagy is known to prevent heightened inflammasome-mediated responses including immunopathology during viral infection^12^. Impaired autophagy, whether at the level of cargo sorting^19^, autophagosome biogenesis^20,21^ or the lysosome^22^, translates into defective mitophagy, which results in the selective activation of the NLRP3 inflammasome by damaged mitochondria-derived stimuli, including reactive oxygen species (ROS)^23^. Autophagy can also degrade inflammasome components, including ASC^24^ and AIM2^25,26^ and has been implicated in the intracellular degradation of pro-IL-1β^27^. However, which mitochondrial quality control effectors are required to control the magnitude of the inflammasome response have not been clearly defined. A recent study reported that RNA viruses activate the mitochondrial fission GTPase dynamin related protein DRP1 to drive mitochondrial damage and activation of the NLRP3 inflammasome^18^, although this has been debated^28,29^.

Here, we explored the function of the Parkinson’s disease-associated mitochondrial serine protease HtrA2 in the inflammasome pathway and report that HtrA2 regulates a mitochondrial quality control mechanism that selectively inhibits ASC-dependent NLRP3 and AIM2 inflammasomes in an agonist-dependent manner. Although initially reported to function in apoptosis^30^, HtrA2 was later demonstrated as an essential effector of mitochondrial protein folding quality control and cell survival^30^. Its catalytic inactivation in mice and humans leads to early-onset fatal infantile neurodegenerative disorder^31,32^, demonstrating that HtrA2 is neuroprotective. Our results show that disruption of the protease activity of HtrA2 (protease loss-of-function) led to impairment of autophagy, sustained accumulation of ASC oligomers and heightened NLRP3 and AIM2-mediated inflammasome responses. Our results confirm the central role of the mitochondria in integrating viral infection signals into regulation of inflammasome signaling^33,34^, and uncover HtrA2 as a non-redundant effector in this process.

## Results

### The protease activity of HtrA2 restricts prolonged NLRP3 inflammasome-induced caspase-1 activity

**Figure 1:**
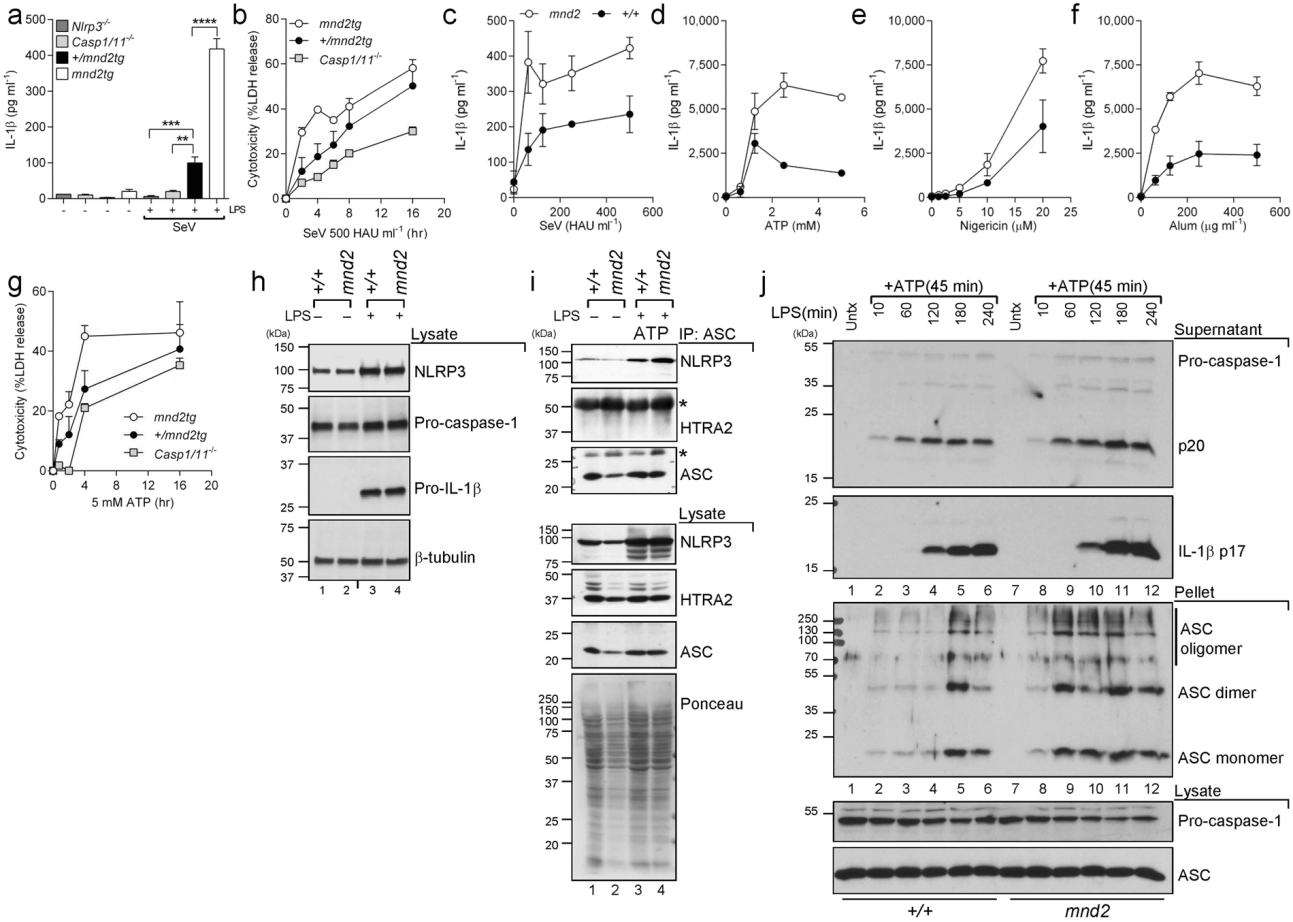
HtrA2 protease activity inhibits the NLRP3 inflammasome. (**a**) IL-1β and (**b**) LDH release (cytotoxicity) in supernatants of LPS (500 ng ml^*−*1^, 3 h)-primed primary BMDMs (from *mnd2tg* and protease-sufficient littermate controls (+/+, +/*mnd2tg*), and infected with SeV (500 HAU ml^−1^) for 18 h. (**c**–**f**) IL-1β secretion by LPS-primed immortalized +/+ and *mnd2* macrophages (derived from 3-week-old non-transgenic mice) treated with the indicated concentrations of inflammasome agonists: (**c**) SeV (18 h), (**d**) ATP (45 min), (**e**) nigericin (30 min), (**f**) Alum (5 h). (**g**) LDH release at the indicated times from LPS-primed *mnd2tg* and protease-sufficient littermate (+/+, +/*mnd2tg*) primary BMDMs treated with ATP (5 mM, 45 min). (**h**) Immunoblot analysis of inflammasome protein levels in lysates from LPS-primed immortalized BMDMs. (**i**) Immunoprecipitation (IP) of endogenous ASC and immunoblot of NLRP3 15 minutes after ATP (5 mM) stimulation in IP or cell lysates of LPS-primed immortalized +/+ and *mnd2* BMDMs. (**j**) Immunoblots showing ASC oligomerization in disuccinimidyl suberate (DSS) cross-linked NP40-insoluble pellets of primary BMDMs, from non-transgenic *mnd2* and *mnd2*/+ littermates (3 weeks old), primed with 500 ng ml^−1^ LPS for the indicated times (min) followed by stimulation with 5 mM ATP for an additional 45 min. Immunoblots of cleaved caspase-1 p20 and IL-1β p17 in culture supernatants of the corresponding samples are shown above the ASC panel; underneath: total pro-caspase-1 and ASC levels in the cell lysates of all samples. Data are representative of two experiments (**c-f**) mean ± s.d.; n = 3 technical replicates per experiment) and of one independent experiment performed once (**a**: mean ± s.e.m.; **b**,**g**: mean ± s.d.; n = 3 mice/genotype, **h**,**i**,**j**). *p < 0.05, **p < 0.01, ***p < 0.001, ****p < 0.0001 Tukey one-way ANOVA post-test. (See also Figure S1).

**Figure 2:**
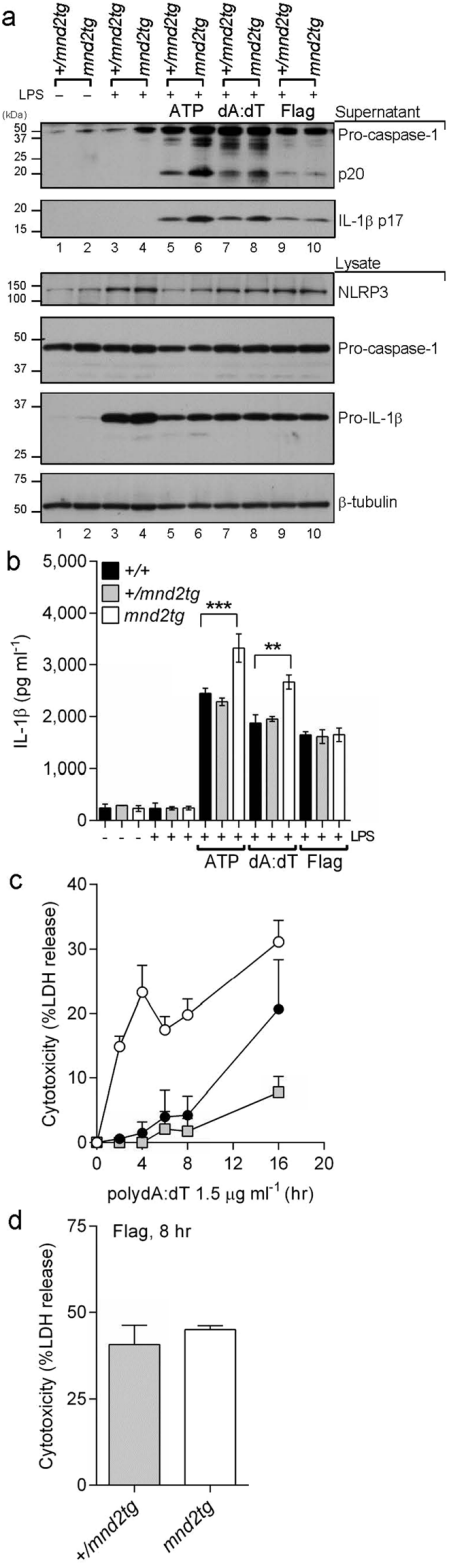
HtrA2 protease activity inhibits the AIM2 but not NLRC4 inflammasome. (**a**,**b**) LPS-primed *mnd2tg* and protease-sufficient littermate (+/+, +/*mnd2tg*) primary BMDMs were treated with ATP (5 mM, 45 min), or transfected with synthetic B-DNA analog (poly dA:dT, 1.8 μg ml^*−*1^; 18 h) or flagellin (1 μg ml^*−*1^, 18 h). Cell supernatants were assayed for (**a**) cleaved caspase-1 p20 and IL-1βp17 by immunoblot. NLRP3, pro-IL-1β and pro-caspase-1 levels were detected in cell lysates by immunoblot. (**b**) IL-1β levels were quantified by ELISA. (**c**,**d**) LDH release at the indicated times from LPS-primed *mnd2tg* and protease-sufficient littermate (+/+, +*/mnd2tg*) primary BMDMs treated with (**c**) polydA:dT (1.5 μg ml^*−*1^) or (**d**) flagellin (1 μg ml^*−*1^). Data are representative of two experiments (**a**,**b**) (**b**: mean ±s.e.m.; n = 3 mice per experiment) and of one independent experiment performed once (**c**,**d**).

To address the role of HtrA2 in inflammasome regulation, we first examined inflammasome responses in bone marrow-derived macrophages (BMDM) from mice harboring the S^276^C *mnd2* catalytic domain mutant allele, which encodes an enzymatically inactive HtrA2 protease^31^. Because HtrA2 protease deficiency leads to neurodegeneration and early postnatal lethality in mice, we also examined BMDMs from viable *mnd2tg* mice, which are deficient in HtrA2 protease activity in all non-neuronal tissues but are rescued from neurological disease by HtrA2 transgene expression in neurons^35^. First, we examined the macrophage NLRP3 inflammasome response using different NLRP3 triggers including infection with Sendai paramyxovirus (SeV). Consistent with a previous report^36^, SeV-elicited IL-1β production was impaired in BMDMs from *Nlrp3*^−/−^ mice or *Casp1*^−/−^ mice harboring a null mutation in *Caspase*-*11* (herein referred to as *Casp1/11*^−/−^ mice) (Fig. 1a). Deficiency in HtrA2 protease activity resulted in increased SeV-elicited IL-1β production (Fig. 1a) and caspase-1-dependent cell death (Fig. 1b) in *mnd2tg* BMDMs relative to protease-sufficient controls. These results were confirmed using BMDMs from the original non-transgenic *mnd2* mice (Fig. 1c). To confirm regulation of the NLRP3 inflammasome by HtrA2, we examined the response of *mnd2* and *mnd2tg* BMDMs to other NLRP3 stimuli. As for SeV, *mnd2* BMDMs treated with ATP (Fig. 1d), nigericin (Fig. 1e) or Alum (Fig. 1f) secreted increased amounts of IL-1β. Similarly, *mnd2tg* BMDMs had increased ATP-induced IL-1β maturation and enhanced caspase-1 proteolysis (Fig. 2a) as well as increased caspase-1-dependent cell death (Fig. 1g). In contrast, TNFα production in these conditions was comparable among genotypes (Supplementary Fig. 1a). To explore the mechanism by which HtrA2 activity impacted the NLRP3 inflammasome, we first examined whether it regulated its priming (signal 1) (which requires ERK and NF-κB-dependent upregulation of NLRP3 and pro-IL-1β gene expression^37^) or its activation (signal 2). A previous report showed that, in microglia, HtrA2 cleaved the mitogen-activated protein kinase (MAPK) kinase MEK1, which dampened ERK1/2 and NF-κB signaling and inhibited microglial activation^38^. To address if HtrA2 regulated the NLRP3 inflammasome priming step through this or other mechanisms in macrophages, we examined the expression of NLRP3 inflammasome components at steady state or after priming with LPS in wild-type, *mnd2* or *mnd2tg* BMDMs. Protein levels of NLRP3, pro-caspase-1, pro-IL-1β and ASC were similar in untreated or LPS-primed macrophages from all genotypes (Figs 1h and 2a), indicating that HtrA2 is dispensable for NLRP3 inflammasome priming. In contrast, we observed enhanced assembly of the NLRP3-ASC complex in *mnd2* macrophages as early as 15 minutes after ATP stimulation (Fig. 1i). Similarly, ASC oligomerization was not only more potent in *mnd2* BMDMs but was also more sustained relative to cells from littermate controls (Fig. 1j). Of note, we did not detect HtrA2 in the NLRP3-ASC complex after ATP stimulation (Fig. 1i and Supplementary Fig. 1b), suggesting that HtrA2 does not inhibit inflammasome activity by directly blocking the NLRP3-ASC interaction. To explore whether the observed increase in NLRP3 inflammasome activation in the absence of HtrA2 protease activity stemmed from differential production of ROS, a reported regulator of the NLRP3 inflammasome^23^, we quantified ROS levels and found them to be similar between *mnd2* and control cells (Supplementary Fig. 1c). In sum, these data show that while HtrA2 protease activity is dispensable for the expression of core inflammasome components, it is necessary to attenuate NLRP3-ASC activation in response to either SeV infection or other stimuli that activate NLRP3 in macrophages.

### The HtrA2 protease selectively inhibits NLRP3 and AIM2 inflammasomes

To explore whether HtrA2 regulates additional inflammasomes besides NLRP3, BMDMs were transfected with either the dsDNA analog polydA:dT or *S. typhimurium* flagellin to activate the AIM2 or NLRC4 inflammasomes, respectively^39^. Levels of secreted IL-1β and caspase-1 processing were increased in LPS-primed *mnd2* and *mnd2tg* BMDMs transfected with dsDNA compared with protease-sufficient BMDMs from littermate control mice (Fig. 2a,b and Supplementary Fig. 2a,b). As for the NLRP3 response, *mnd2tg* BMDMs exhibited increased caspase-1-dependent cell death to dsDNA relative to controls (Fig. 2c). In contrast, *mnd2tg* BMDMs transfected with flagellin showed similar levels of active caspase-1, IL-1β production and cell death compared to control BMDMs (Fig. 2a,b,d), suggesting that HtrA2 is not involved in modulating the NLRC4 inflammasome. Collectively, these data demonstrate that HtrA2 inhibits the NLRP3 and AIM2 inflammasome pathways, likely through a common mechanism.

### HtrA2 inhibits inflammasome activity indirectly through modulation of autophagy flux

**Figure 3:**
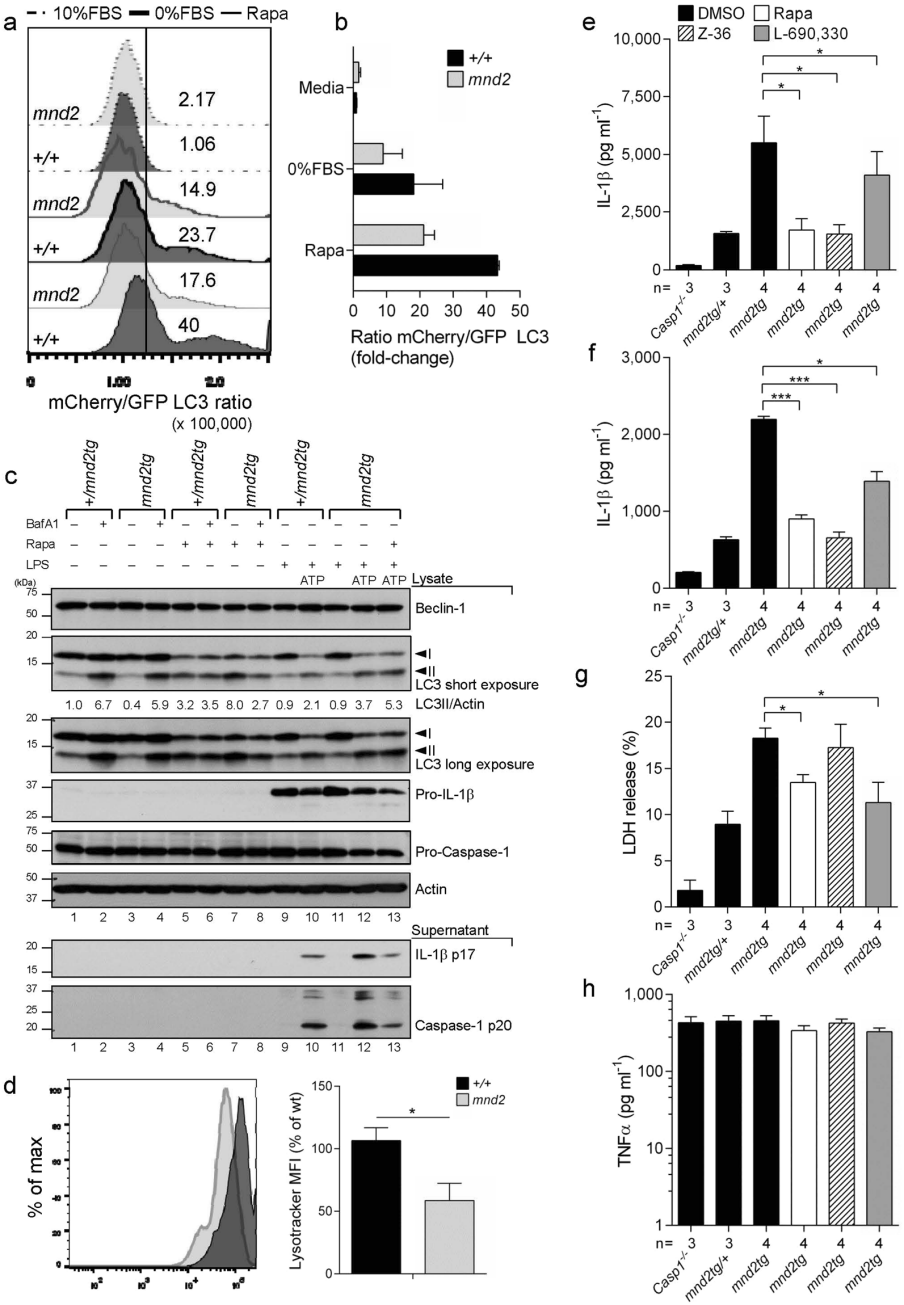
HtrA2 controls autophagy flux-dependent inhibition of the inflammasome. (**a**) Representative differences and (**b**) quantification of autophagy flux as measured by the flow cytometric ratio of mCherry/GFP LC3 in transduced *mnd2* and wild-type (+/+) immortalized BMDMs cultured for 16 h in complete media (dashed line), serum deprivation (heavy solid line) or rapamycin (5 μM) (solid line). (**c**) Immunoblot analysis for Beclin-1, LC3, IL-1β, caspase-1, cytochrome C and actin from *mnd2tg* primary BMDMs pre-treated with rapamycin (5 μM) for 1.5 h before ATP (5 mM, 2 h) treatment after LPS (0.5 μg ml^−1^, 4 h) stimulation; +/*mnd2tg* littermates serve as control. (**d**) Flow cytometry quantification of lysotracker mean fluorescence intensity (MFI) from the indicated genotypes. (**e-f**) IL-1β secretion quantified by ELISA in supernatants from LPS-primed *mnd2tg* BMDMs pre-treated with autophagy inducers rapamycin (3 μM), Z-36 (BCL-XL inhibitor, 3 μM) and L-690,330 (IMPase inhibitor, 100 μM) for 1.5 h before (**e**) ATP (5 mM, 45 min) treatment or (**f**) poly dA:dT transfection (1.5 μg ml^−1^, 18 h);+/*mnd2tg* littermates and *Casp1/11*^−/−^ serve as controls. (**g**) LDH release and (**h**) ELISA for TNFα in supernatants from BMDMs treated as in **e**. Data are representative of two experiments (**a**,**b**: mean $\pm$ s.d., n = 2–3 replicates per experiment; **c**,**e**,**g**,**h**: mean ±s.e.m., n = 3–4 mice per experiment), pooled from two independent experiments (**d**: mean $\pm$ s.em., n = 2 replicates per experiment), and one experiment (**f**; mean ±s.e.m.; n = 3–4 mice). *p < 0.05, ***p < 0.001 (Mann-Withney test (**d**); Dunnet one-way ANOVA post-test (**e**, **f**, **g**)). (See also Figure S3).

Since HtrA2 was reported to control autophagy^40^, a process implicated in inhibiting both NLRP3 and AIM2 inflammasomes^17^, we interrogated the role of autophagy modulation by HtrA2 in inflammasome inhibition. We expressed the autophagosome marker mCherry-GFP-LC3 in *mnd2* and wild-type BMDMs by retrovirus-mediated transduction and used FACS to measure relative levels of autophagic flux after treatment with rapamycin or after serum deprivation. Cells with increased flux emit less green fluorescence owing to the acidic environment of the autophago-lysosome and this is measured as an increase in the mCherry/GFP ratio^41^. Wild-type macrophages treated with rapamycin showed increased mCherry/GFP ratio indicating that autophagosomes were converting into autolysosomes (Fig. 3a,b). This was supported by enhanced phosphatidylethanolamine (PE) conjugation of LC3 as detected by changes in LC3-II levels in response to rapamycin (Fig. 3c, lanes 5 versus 1). In contrast, *mnd2* macrophages showed marginal increase in the mCherry/GFP ratio, suggesting that rapamycin- or serum deprivation-induced autophagy flux is greatly attenuated in the absence of functional HtrA2 proteolysis (Fig. 3a,b). Consistently, *mnd2tg* BMDMs showed increased accumulation of LC3-II following rapamcyin compared to controls (Fig. 3c, lane 7 versus 5). As expected, bafilomycin A1 (BafA1), a compound that prevents lysosomal degradation, elevated LC3-II levels at steady state (Fig. 3c, lanes 1–4). In contrast, BafA1 did not enhance LC3-II accumulation in rapamycin-treated *mnd2tg* BMDMs (Fig. 3c, lane 8 versus 7), pointing to impaired autophagy flux rather than increased autophagy induction in the absence of HtrA2 protease activity. As with rapamycin, ATP stimulation of LPS-primed macrophages induced autophagy in control (Fig. 3c, lane 10 versus 9) and *mnd2tg* (Fig. 3c, lane 12 versus 11) BMDMs, with enhanced accumulation of LC3-II in *mnd2tg* BMDMs compared to control cells (Fig. 3c, lane 12 versus 10). Although basal autophagy flux was similar between genotypes (Fig. 3a,b), confocal imaging revealed that *mnd2* macrophages had less GFP-LC3 puncta constitutively and fewer cells with greater than 3 strong GFP-LC3 puncta per cell with addition of chloroquine to block lysosomal degradation (Supplementary Fig. 3a-c). Combined with reduced basal LC3-II levels (Fig. 3c, lane 3 versus 1), these data indicate a decreased autophagosomal content at baseline, as previously reported for HtrA2-deficient mouse embryonic fibroblasts (MEFs)^40^. Furthermore, using the LysoTracker probe, we recorded a ∼50% decrease in lysososome abundance in *mnd2* macrophages as compared to wild-type cells (Fig. 3d). Our data indicate that the need for HtrA2 protease activity in autophagy flux is most apparent under stress conditions, such as inflammasome activation, and not under normal growth conditions.

Based on these results, we tested whether increased inflammasome function in *mnd2tg* BMDMs is due to impaired autophagy flux. For that, LPS-primed *mnd2tg* and protease-sufficient control BMDMs were treated with rapamycin shortly prior to ATP stimulation or dsDNA transfection. In agreement with our hypothesis, rapamycin not only corrected the exaggerated ATP-induced inflammasome responses of *mnd2* macrophages, including IL-1β maturation (Fig. 3c,e) and cell death (Fig. 3g), but also reduced IL-1β production in protease-sufficient control BMDMs (Supplementary Fig. 3d), in agreement with previous reports^24,42^. Similarly, rapamycin restored excessive AIM2*-*induced inflammasome activity to normal levels in *mnd2tg* BMDMs treated with dsDNA (Fig. 3f), indicating that improved autophagy functions reduce the exacerbated response of not only NLRP3 but also AIM2 inflammasome in HtrA2 protease-deficient cells. We further confirmed this by using L690–330 and Z36, which activate autophagy in an mTOR-independent manner, but through distinct mechanisms^43,44^ (Fig. 3e–f). TNFα secretion in supernatants from BMDMs treated with autophagy inducers and ATP was unchanged (Fig. 3h). To explore if the role of HtrA2 in autophagy and the turnover of ASC-inflammasome complexes is conserved in human cells, we used ASC reporter HEK293T fibroblasts^45^ and stably depleted by shRNA HtrA2 or the autophagy protein ATG5. First, we examined autophagy flux regulation by HtrA2, and found that as for murine *mnd2* and *mnd2tg* macrophages, depletion of human HtrA2 similarly resulted in impaired autophagy flux following rapamycin or serum starvation, as assessed using the mCherry-GFP-LC3 reporter (Supplementary Fig. 3e). Second, we quantified the accumulation of ASC oligomers using a flow cytometric assay that detects changes of ASC distribution within a cell^46^. As expected, inhibition of autophagic flux using BafA1 increased the content of ASC oligomers in response to SeV infection (Supplementary Fig. 3f). Next, we performed cycloheximide-chase analysis of formed ASC complexes in SeV infected cells. Blocking protein synthesis with cycloheximide at 18 hours post-infection revealed slower ASC oligomer turnover in HtrA2- or ATG5-depleted cells than in control cells (Supplementary Fig. 3g). Collectively, these data suggest that defects in autophagy flux and lysosomal degradation account at least in part for the accumulation of ASC-containing inflammasomes and overactive caspase-1 in cells lacking HtrA2.

### HtrA2 regulates caspase-1-dependent inflammatory responses *in vivo*

**Figure 4:**
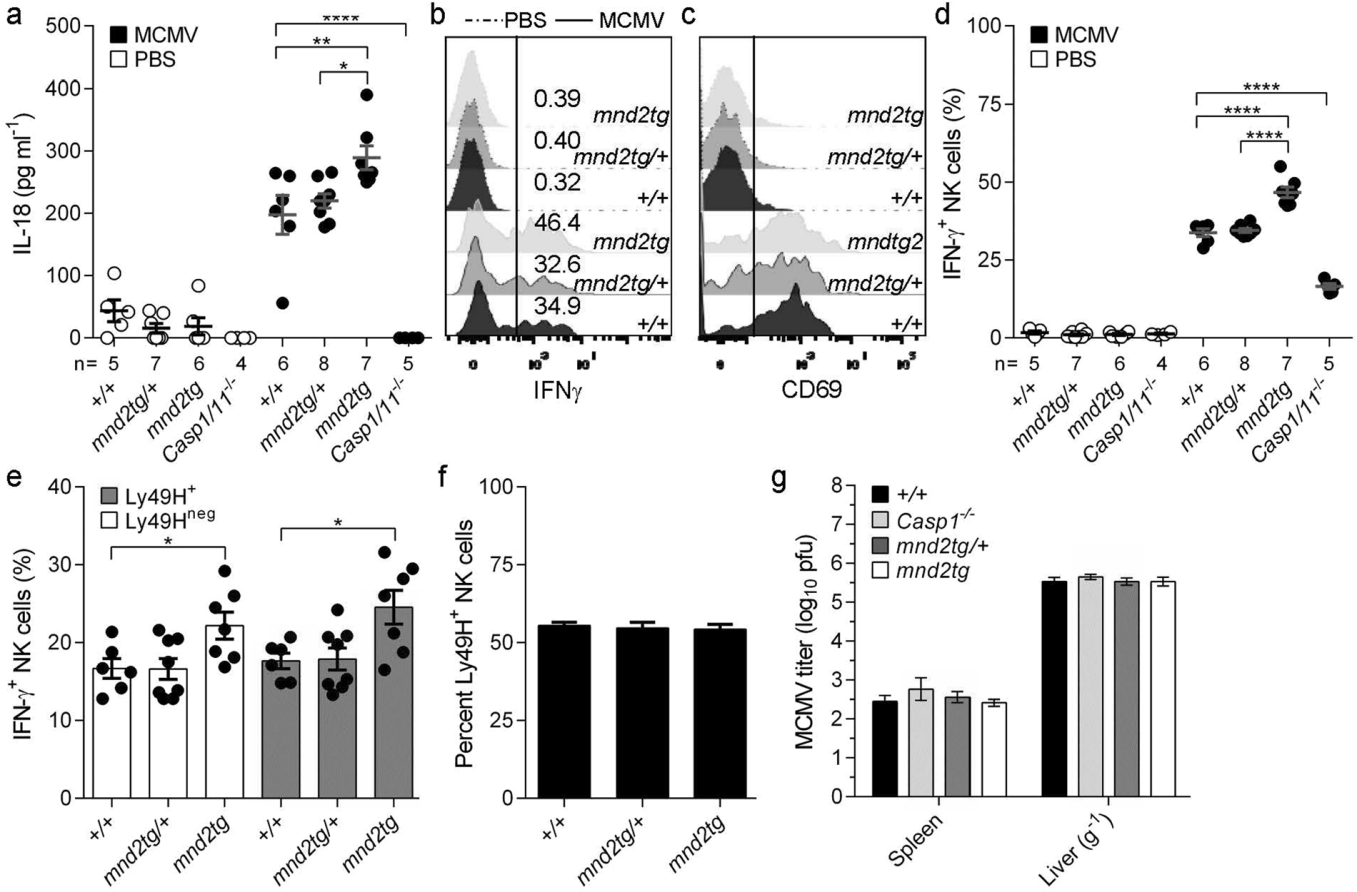
HtrA2 controls caspase-1-dependent cellular inflammatory responses to MCMV. *Mnd2tg*, littermate controls (+/+, +/*mnd2tg*) and *Casp1/11*^*−/−*^ mice were injected i.p. with MCMV (Smith strain, 6,000 pfu/mouse) or PBS (mock control, 200 μl/mouse). (**a**) Serum IL-18 concentrations quantified by ELISA 40 h post-infection with MCMV or in PBS mock-infected mice. Representative (**b**) intracellular expression of IFNγ and (**c**) surface expression of CD69, assessed by flow cytometry (gated on NK1.1^+^CD3^−^TCRβ^−^ viable splenocytes cultured 4 h in medium containing brefeldin A (5 μg ml^−1^) and monensin (5 μg ml^−1^) without additional stimuli; (*x* axis, fluorescence intensity; *y* axis, percentage of maximum); dashed curves indicate NK cells from uninfected mice. (**d**) Quantification of NK-derived IFN-γ responses at 40 h post-infection. (**e**) Proportions of splenic NK cells expressing surface Ly49H and intracellular IFN-γ. (**f**) Percentages of splenic Ly49H^+^ NK cells prior to infection. (**g**) MCMV titers in spleen and liver at 40 h post-infection. Symbols indicate individual mice. All data represent two independent experiments pooled (mean ± s.e.m., n = 4–8 mice per genotype). *p < 0.05, **p < 0.01, ****p < 0.0001 (Tukey (**a**,**d**) or Dunnet (**e**) one-way ANOVA post-test). (See also Figure S4).

To determine the role of HtrA2 in the regulation of inflammasome responses *in vivo*, we chose a mouse model of acute MCMV infection. In this model, caspase-1-dependent IL-18 is produced downstream of AIM2 activation and is important for the generation of the early MCMV-induced production of IFNγ by splenic NK cells^3^. However, in contrast to IL-12, IL-18 is dispensable for MCMV viral control and host survival^47^. Thus, this model is useful to examine inflammasome responses independently of effects on viral loads. We infected viable *mnd2tg* mice and littermate controls with 6 × 10^3^ pfu/mouse by intraperitoneal injection. Consistent with previous reports^3,48,49^, infection with MCMV for 40 hours induced IL-18 in the serum (Fig. 4a) and led to MCMV-specific IFNγ production by splenic NK cells in wild-type controls, whereas these responses were dampened in *Casp1/11*^−/−^ mice (Fig. 4a,d). In contrast, *mnd2tg* mice exhibited heightened serum IL-18 levels 40 hours post-infection compared to littermate controls, concomitant with larger increases in the frequency of IFNγ-producing splenic NK cells (Fig. 4a,b,d). These early inflammasome responses were independent of differences in viral loads, as viral titers were equivalent in all genotypes tested (Fig. 4g). Ly49H^+^ NK1.1^+^ cells make up to 50% of NK cells in C57BL/6 mice and are essential in MCMV infection^50,51^. Whereas *mnd2tg* mice (C57BL/6 background) displayed comparable baseline Ly49H-expressing NK1.1^+^CD3^−^ frequencies (Fig. 4f) and comparable post-infection spleen weights (Supplementary Fig. 4a), they had significantly higher IFNγ-producing Ly49H^+^ NK cell frequencies relative to control littermates (Fig. 4e). This response was specific to MCMV and was not due to NK cell intrinsic defects, since PMA and ionomycin stimulation induced comparable numbers of IFNγ^+^ NK cells regardless of the HtrA2 protease functional status (Supplementary Fig. 4b). There was similar expression of the activation marker CD69 on NK cells within all genotypes (Fig. 4c, Supplementary Fig. 4c,d), indicating that the observed differences could not be attributed to altered numbers of NK cells responding to MCMV. These results indicate that under comparable conditions of MCMV infection, mice with HtrA2 loss-of-function develop a stronger early caspase-1-dependent inflammatory response *in vivo*, consistent with HtrA2 being a negative regulator of the inflammasome pathway.

## Discussion

Inappropriate inflammasome signaling has been linked to a variety of inflammatory disorders, including viral and neurodegenerative diseases^14,52–54^. Given the evolutionary conservation of the HtrA family of proteases and its role in protein metabolism and cell fate^55^, it is unlikely that HtrA2 functions as a dedicated or direct inhibitor of ASC-dependent inflammasomes. In fact, it is not found within the inflammasome complex. Our analysis shows that the HtrA2 S^276^C protease inactive mutant had no effect on LPS-induced pro-inflammatory gene expression or inflammasome priming in activated macrophages. Instead, our data suggest that HtrA2 reduces inflammasome activity, in part, by promoting the later stages of autophagy during stress conditions, thereby ensuring the turnover of ASC oligomers. We demonstrate with a range of assays, including studies of flux using a mCherry-GFP-LC3 reporter and lysosome probes and endogenous LC3-II analysis in the presence of the lysosome inhibitor BafA1 that the protease activity of HtrA2 is necessary for the maturation of autophagosomes into autolysosomes. These findings do not exclude the possibility that the defect in autophagy may translate into impaired mitophagy, which might explain the overstimulation of NLRP3 in macrophages harboring the HtrA2 protease inactive S^276^C *mnd2* mutant. However, defects in mitophagy do not account for the observed AIM2 hyperactivation in these cells. Furthermore, our results indicate that *mnd2* macrophages do not overproduce ROS before or after inflammasome stimulation, and do not display increased mitochondrial damage in the conditions tested. This is in contrast to phenotypes reported for mitophagy-impaired macrophages^19,56^, but is in line with studies showing that wild-type HtrA2 inhibits mitophagy by cleaving the two mitophagy promoting E3 ligases MUL1 and Parkin^57–59^. Our observations that rapamycin and other autophagy inducers corrected the excessive inflammasome activity in HtrA2 S^276^C mutant cells suggest that the effect of HtrA2 on inflammasome functions is largely autophagy-dependent. The observations that the anti-inflammatory IL-1 family cytokine IL-37 inhibits mTOR, induces autophagy and dampens the NLRP3 inflammasome^60^, further highlights the regulation of the inflammasome pathway by autophagy, and points to a potential therapeutic role of IL-37 in mitochondria-related human immunopathologies.

Together, our observations are consistent with emerging data that show that loss of mitochondrial quality control pathways can disrupt lysosomal proteolytic activity, thus impairing autophagic flux, exacerbating inflammatory responses and increasing cell death^61,62^. Moreover, recent studies support the direct contribution of lysosomal homeostasis to the negative regulation of the inflammasome. A similar phenotype of exaggerated caspase-1 activity and IL-1β cleavage was reported in human *GBA1*^*N370S*^ macrophages, which present lysosomal dysfunction due to deficient glucocerebrosidase activity^22^. Although autophagy can degrade inflammasome components^19,24–27^, it is also required for IL-1β secretion from living cells through a TRIM16-Sec22b secretory autophagic pathway, which is itself modulated by lysosome function^63,64^. It is tempting to hypothesize that by coupling mitochondria-lysosomal crosstalk with inflammasome function, cells can use their metabolic fitness to allow flexible adjustment of inflammasome activation.

*In* vivo, we show that HtrA2 limits IL-18-dependent early inflammatory responses to MCMV (40 hours post-infection). A previous study implicated HtrA2 in cytomegalovirus-associated programmed cell death, a process occurring late in the infection (day 6–10 post-infection) and antagonized to some extent by the viral protein vMIA^65^. It is plausible that during cell death, e.g. at later time points in the infection, HtrA2 exerts functions distinct from its physiological role in mitochondrial quality control and autophagy, as previously discussed^30^.

Our results suggest that HtrA2 regulation of inflammasomes seems to be a conserved mechanism that counteracts inflammation to unrelated viruses downstream of the agonist-induced nucleating step. The independence of the NLRC4 inflammasome from HtrA2 regulation remains unclear. A plausible reason is that NLRC4 can recruit and activate pro-caspase-1 independently of ASC^66^. An alternative reason could be that NLRC4 can actively inhibit autophagy^67–69^. Thus, the overall influence of HtrA2 on inflammasome activation will be dependent on potentially complex interactions between degradative lysosomal pathways, secretory autophagy pathways, and on the specific ligands involved. The unexpected role of HtrA2 in modulating autophagy flux to control inflammasome responses provides a new framework to understand mitochondria-related immunopathologies in humans, including neurodegeneration.

## Methods

### Mice

All mice were fully on the C57BL/6J background and were bred and maintained at McGill University Life Sciences Centre specific pathogen-free animal facility. *Nlrp3*^−/−^, *Casp1/11*^−/−^ (deficient for caspase-1 and -11) and C-section re-derived *HtrA2*^mnd2^ transgenic mice (referred to here as *mnd2tg*) have been reported^35,70^. Animals were used when 8–10 weeks old, except for non-transgenic *mnd2* mice that were used at 3 weeks of age. All animal experiments were in accordance with the regulatory guidelines of the Canadian Council on Animal Care and were approved by the McGill University Animal Care and Ethics Committee.

### Virus infection and tissue sampling

MCMV (Smith strain) stocks were prepared from salivary glands of 3-wk old BALB/c mice, aliquoted and stored at −80 °C. Age- and sex-matched mice were infected i.p. with 6 × 10^3^ pfu/mouse in 200 µl of PBS or mock-infected with PBS alone. Blood for serum isolation (by cardiac puncture), spleen and liver were collected from infected mice at 40 hours post-infection for ELISA, viral plaque assays and FACS analysis. Spleens were mechanically disrupted and passed through 70 µm cell strainers, following which 1/4 of the sample was used for plaque assay and the rest was treated with ACK lysis buffer to remove red blood cells. The resulting single cell suspensions were used for stimulations and FACS analysis.

### Virus titration by plaque forming unit assay

Serial dilutions of spleen and liver homogenates were performed in PBS and used to infect confluent MEF cells for 1 hour at 37 °C, 5%CO_2_. Infection was pursued for 3 days and plaques were visualized by crystal violet staining.

### BMDM culture and stimulation

Femurs and tibias of *mnd2tg* littermates, *mnd2*, wild-type, *Nlrp3*^−/−^ and *Casp1/11*^−/−^ mice were flushed to obtain marrow. Cells were re-suspended at 4 × 10^6^ cells per 10 cm non-tissue culture-treated dishes and differentiated into macrophages (BMDM) at 37 °C 5% CO_2_ for 7 days in RPMI-1640 medium (containing 10% heat-inactivated FBS, 2 mM glutamine, 100 μg/ml penicillin/streptomycin, 50 μM 2-mercaptoethanol, 1% non-essential amino acids) supplemented with 25% M-CSF conditioned median on days 0, 3 and 5. BMDMs were adhered overnight in 96-well plates (0.75 × 10^5^ cells), in 24-well plates (5 × 10^5^ cells) or 6-well plates (1 × 10^6^ cells) and primed for 4 hours with 500 ng ml^−1^ ultra-pure LPS in OPTI-MEM (Life Technologies) or otherwise indicated. For inflammasome stimulations, primed cells were infected with SeV at 500 HAU ml^−1^ or treated with 5 mM ATP, 1.8 μg ml^−1^ dsDNA plus 0.1% v/v Lipofectamine 2000 (Life Technologies), 1 μg ml^−1^ flagellin plus 0.25% v/v DOTAP (Roche), 500 μg ml^−1^ Alum, 2 μg ml^−1^ 5′pppRNA plus 0.1% v/v Lipofectamine 2000 for the indicated periods (or otherwise) indicated in figure legends. Cleaved IL-1β was measured by ELISA in culture supernatants. Cytotoxicity was measured by LDH release using a CytoTox 96 Non-Radioactive Cytotoxicity assay (Promega). For pharmacological rescue of autophagy, autophagy inducer drugs were added to primed BMDMs 1.3 hours before stimulation with ATP or transfection with dsDNA. Immortalized BMDMs were transduced with MMLV-based retrovirus encoding mCherry-EGFP-LC3 and selected on day 2 by addition of puromycin (5 μg ml^−1^).

### Confocal microscopy

Immunofluorescence slides were analyzed on an inverted Zeiss LSM510 confocal microscope (20 × 0.75 and 63 × /1.4 Plan-Apochromat or 40 × /1.3 Pan-Neofluar objectives). ImageJ 1.46 (National Institute of Health) was used for processing of images before cropping to emphasize the main point of the image when appropriate; processing was limited to background subtraction, brightness/contrast adjustments and pseudo colors addition to facilitate the visualization/interpretation of the results. BMDMs plated on chamber slides overnight were primed with 500 ng ml^−1^ LPS for 6 h, then stimulated with 5 mM ATP for 15 minutes. After stimulations, cells were fixed with 4% formaldehyde for 10 minutes, permeabilized with 0.1% Triton X-100, and the slides were blocked with PBS buffer containing 3% BSA. Cells were immunostained with anti-ASC, anti-HtrA2 (V-17, Santa Cruz) and Alexa-Fluor 488 and 647 conjugated secondary antibodies for colocalization assay during inflammasome assembly in BMDMs.

### Immunoblotting

Cells were homogenized and lysed in B150 buffer (20 mM Tris-HCl pH 8.0, 150 mM KCl, 10% glycerol, 5 mM MgCl_2_, 0.1% NP-40 supplemented with a protease inhibitor cocktail (11836153001; Roche), followed by boiling in Laemmli sample buffer for examination by immunoblot. For cleavage studies (caspase-1 p20, and IL-1β p17), 7% of individual culture supernatants was loaded and resolved on gel. For endogenous co-immunoprecipitation studies, whole cell lysates (1 mg protein) in B150 were cleared by incubation with protein G sepharose beads (P3296; Sigma-Aldrich), incubated with anti-ASC antibody for 2.5 hours at 4 °C and protein G sepharose beads were added for an additional hour. Immunoprecipitates were eluted by boiling in Laemmli buffer, electrophoresed on 10% SDS polyacrylamide gels and processed for immunoblot analysis using antibodies to mouse anti-NLRP3. ASC polymerization was assayed in disuccinimidyl suberate cross-linked NP-40 insoluble pellets following SDS-PAGE fractionation as described before^27^.

### Flow cytometry assays

Measurement of NK responses was performed using a modified version of published methods^71^. Leukocytes isolated from spleen of infected mice were examined for viability by trypan blue exclusion (typically ≥95%) and 2 × 10^5^ cells/well was plated in 96-well V bottom plates. Brefeldin A (5 μg ml^−1^ final concentration) and monensin (5 μg ml^−1^) was added for 4 hours at 37 °C. In certain conditions, leukocytes were stimulated with PMA (50 ng ml^−1^) and ionomycin (375 ng ml^−1^) in the presence of Brefeldin A and monensin for 4 hours. Following isolation and stimulations, cells were surface stained with the appropriate predetermined concentrations of fluorochrome-labeled antibodies and the amine reactive viability dye Live/Dead fixable stain (GhostDye Red780, Tonbo Biosciences) for 20 min in the dark at 4 °C. After intracellular staining for IFNγ, cells were fixed in PBS containing 1% formaldehyde and stored at 4 °C in the dark until FACS analysis (performed within 12 h). Data were acquired on a Fortessa instrument (BD Biosciences) equipped for the detection of 18 fluorescent parameters including mCherry (yellow laser line 562 nm). Data analysis was performed using FACS DiVa version 6.0 software or FlowJo version 10.0. After setting of singlet, lymphocyte gates and exclusion TCRβ^+^CD3^+^ cells, viable splenic NKs were defined as NK1.1^+^ cells and analyzed for Ly49H, CD69 and IFNγ expression. Autophagy flux was acquired as the ratio of mCherry/GFP and ASC-GFP oligomer formation as GFP-H/GFP-A.

### Statistical analysis

Statistical analysis was performed using GraphPad Prism software v5.0. Data are represented as mean ± standard deviation or standard error of the mean.

## Electronic supplementary material

Supplemental information
