# Supplementary material for: The mitochondrial protease HtrA2 restricts the NLRP3 and AIM2 inflammasomes
Source: Sci Rep. 2018 May 31;8:8446. doi: 10.1038/s41598-018-26603-1 (PMC5981608; doi:10.1038/s41598-018-26603-1)
Supplement: Supplementary file 1 — Supplemental information [file 41598_2018_26603_MOESM1_ESM.pdf]

The mitochondrial protease HtrA2 restricts the NLRP3 and AIM2 inflammasomes

Ian Gaël Rodrigue-Gervais[1,2*], Karine Doiron[1], Claudia Champagne[1], Lindsey Mayes[3], Gabriel André Leiva-Torres[4], Paulin Jr Vanié[2], Todd Douglas[5], Silvia M. Vidal[4], Emad Alnemri[3] and Maya Saleh[1,5,6*]

Supplemental information

Supplemental Methods

Reagents.

For immunoblotting: anti-mouse caspase-1 p20 (B4B, Genentech), anti-IL1β (AF401NA, R&D Systems), anti-NLRP3 (Cryo2, AdipoGen), anti-ASC (N-15R, Santa Cruz), anti-β-actin (AC-15, Sigma) or anti-tubulin (H-235, Santa Cruz); anti-Beclin 1 (D40C5) and anti-LC3 (2775) (Cell Signaling Technology). The following DuoSet ELISA kits from R&D were used: anti-mouse IL-1β (DY401) and anti-human IL-1β (DY201). Ultrapure LPS (from *E. coli* strain 0111:B4) was from Invivogen. Brefeldin A, saponin, PMA, ionomycin, ATP, nigericin, rapamycin, Bcl-XL inhibitor Z36, Bafilomycin A1, chloroquine and cycloheximide were all from Sigma-Aldrich. Imject Alum adjuvant was from ThermoFischer Scientific. Inositol monophosphatase inhibitor L690-330 was from Tocris-Bioscience. The following plasmids were obtained directly from Addgene: pBabe mCherry-EGFP-LC3 (22418) and pCL-Eco (12371). The following antibodies or reagents were used for flow cytometry: anti-CD3-PerCPCy5.5 (145-2C11), anti-TCRβ-PerCPCy5.5 (H57-597), anti-CD69-PeCy7 (H1.2F3), anti-Ly49H (all from eBioscience); anti-NK1.1-APC (PK136), anti-IFNγ-PECy7 (XM1.2) (all from BD Biosciences); Annexin V-CF405M, MitoView (Biotium); LysoTracker Deep Red, MitoTracker Red CMXRos (Life Technologies).

Supplemental Figure Legends

Figure S1. Related to Figure 1. *HtrA2 protease regulates the NLRP3 inflammasome*.

(**a**) TNFα secretion in supernatants from LPS-primed *mnd2tg* and protease-sufficient littermate (+/+, +*/mnd2tg*) primary BMDMs treated with ATP (5 mM, 45 min), transfected with synthetic B-DNA analog (poly dA:dT, 1.8 μg ml^{-1}; 18 h) or flagellin (1 μg ml^{-1}, 18 h), or infected with SeV (500 HAU ml^{-1}, 18 h).

(**b**) HtrA2 localization relative to ASC after ATP stimulation by confocal microscopy.

(**c**) Amounts of ROS by FACS in immortalized BMDMs treated as in Fig. 1i.

Data are representative of two experiments (**a**: mean ± s.e.m.; n=3 mice per experiment) and one independent experiment performed once (**b**, **c**).

Figure S2. Related to Figure 2. *HtrA2 protease regulates the AIM2 but not NLRC4 inflammasome*.

(**a**) Cleaved caspase-1 p20 and IL-1β p17 by immunoblot of culture supernatants of LPS-primed *mnd2* and +/+ immortalized BMDMs treated with ATP (5 mM, 45 min) or transfected with poly dA:dT (1.8 μg ml^{-1}; 18 h) or flagellin (1 μg ml^{-1}, 18 h).

(**b**) IL-1β secretion by LPS-primed immortalized +/+ and *mnd2* BMDMs treated with the indicated concentrations of polydA:dT (18 h).

Figure S3. Related to Figure 3. *HtrA2 controls Autophagy flux*.

(**a**) LC3 confocal imaging, (**b**) baseline number of GFP-LC3 puncta and (**c**) frequency of cells with greater than 3 strong GFP-LC3 puncta per cell in LC3-GFP transduced immortalized +/+ or *mnd2* BMDMs cultured in the absence or presence of chloroquine (50 μM, CQ).

(**d**) IL-1β secretion in protease-sufficient BMDMs treated with rapamycin as in Fig. 3c.

(**e**) mCherry/GFP LC3 ratio following rapamycin stimulation in control, HtrA2- or ATG5-depleted HEK-293T cells.

(**f**) ASC oligomerization in response to SeV infection in HEK-293T cells following inhibition of autophagic flux using BafA1 measured by FACS.

(**g**) Cycloheximide-chase analysis of formed ASC complexes in SeV infected control, HtrA2- or ATG5-depleted cells.
Data are representative of two independent experiments (**e**: n>250,000 cells per condition; **g**: mean ± s.d.; n=2 technical replicates per experiment), and independent experiments performed once (**a**,**b**,**c**: mean ± s.e.m., n> 40 cells per condition; **d**: mean ± s.e.m., n=3 mice; **f**: n>500,000 cells per condition). **** $p < 0.0001$ (Tukey one-way ANOVA post-test (**b**, **c**)).

Figure S4. Related to Figure 4. *MCMV infection supporting data.*
(**a**) Spleen weights 40 h post-infection with MCMV.
(**b**) PMA and ionomycin-induced IFNγ in NK cells by intracellular FACS.
(**c, d**) Percentages of splenic $CD69^+$ NK cells and (**d**) CD69 MFI at 40 h post-infection with MCMV.
All data represent two independent MCMV infections pooled (mean ± s.e.m., n=4-8 mice per genotype).

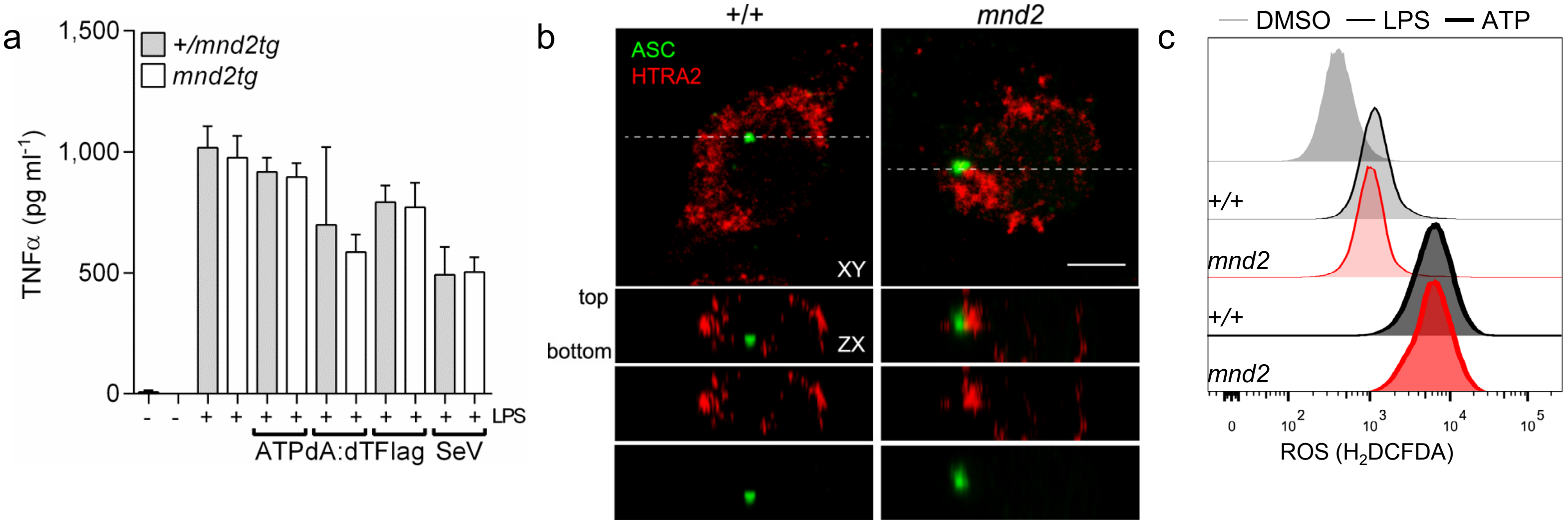
a
1,500
1,000
500
0
TNFα (pg ml-1)
+/mnd2tg
mnd2tg
LPS
ATP
dA:dT
Flag
SeV
b
+/+
mnd2
ASC
HTRA2
XY
ZX
top
bottom
c
DMSO
LPS
ATP
+/+
mnd2
ROS (H2DCFDA)

Figure S1

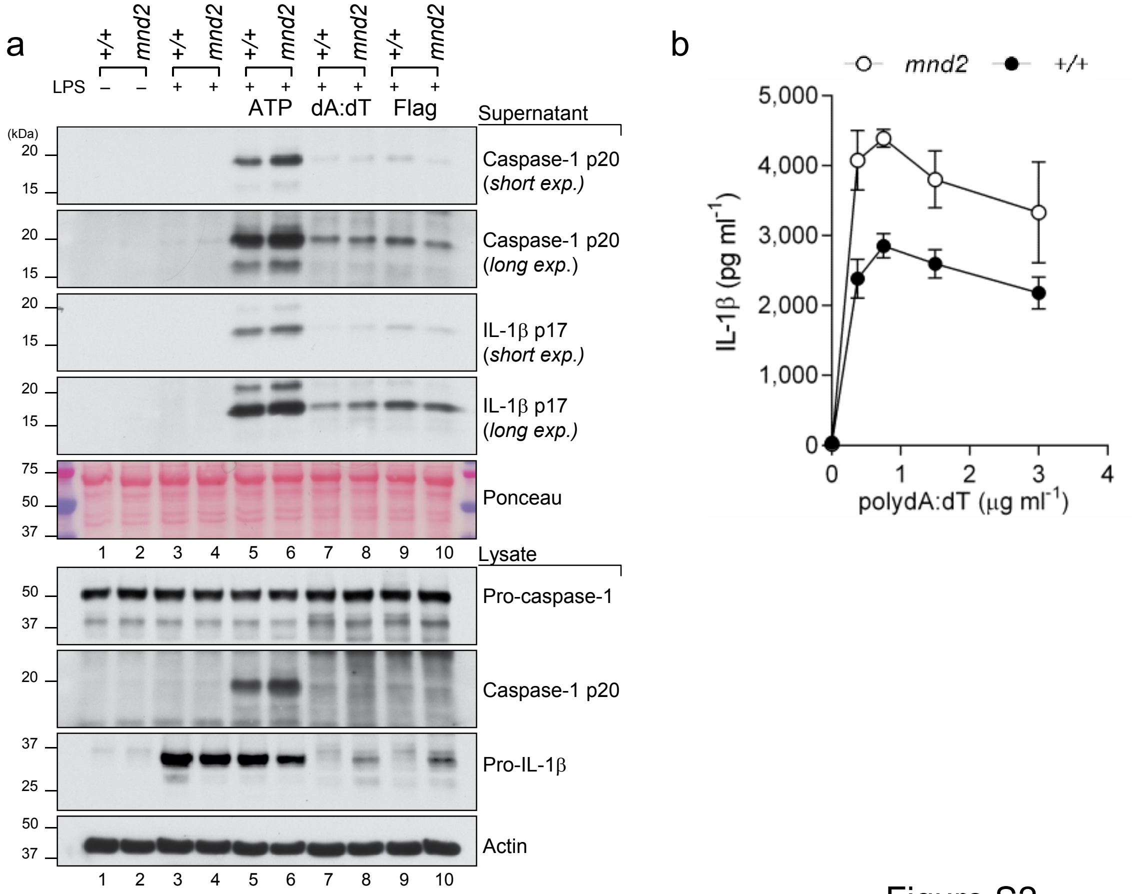
a
+/+
mnd2
LPS
ATP
dA:dT
Flag
Supernatant
(kDa)
Caspase-1 p20
(short exp.)
Caspase-1 p20
(long exp.)
IL-1β p17
(short exp.)
IL-1β p17
(long exp.)
Ponceau
Lysate
Pro-caspase-1
Caspase-1 p20
Pro-IL-1β
Actin
b
mnd2
+/+
IL-1β (pg ml-1)
polydA:dT (μg ml-1)

Figure S2

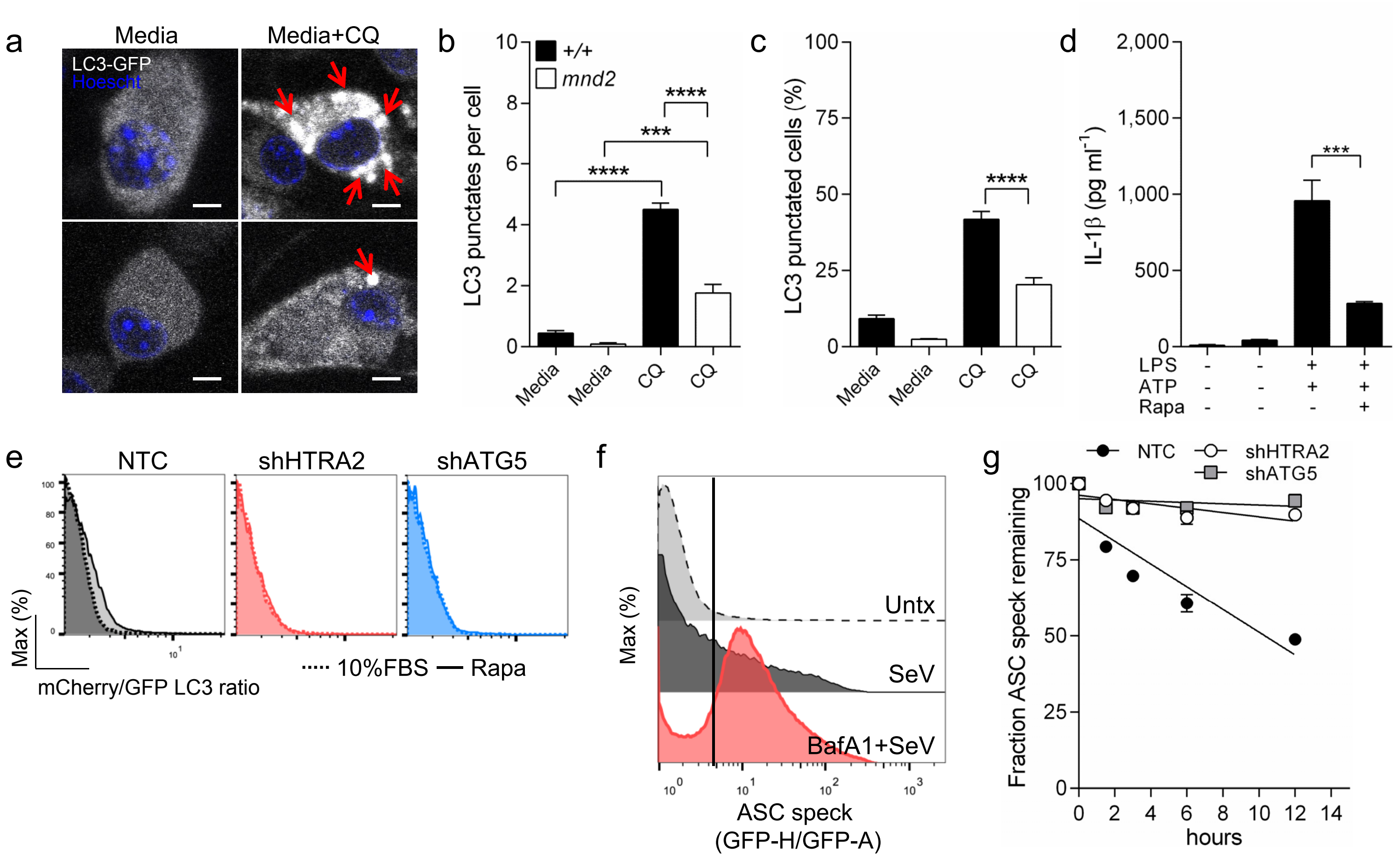
a
Media
Media+CQ
LC3-GFP
Hoescht
b
LC3 punctates per cell
+/+
mnd2
****
***
****
Media
Media
CQ
CQ
c
LC3 punctated cells (%)
****
Media
Media
CQ
CQ
d
IL-1β (pg ml^{-1})
***
LPS - - + +
ATP - - + +
Rapa - - - +
e
NTC
shHTRA2
shATG5
Max (%)
mCherry/GFP LC3 ratio
10%FBS
Rapa
f
Max (%)
Untx
SeV
BafA1+SeV
ASC speck
(GFP-H/GFP-A)
g
NTC
shHTRA2
shATG5
Fraction ASC speck remaining
hours

Figure S3

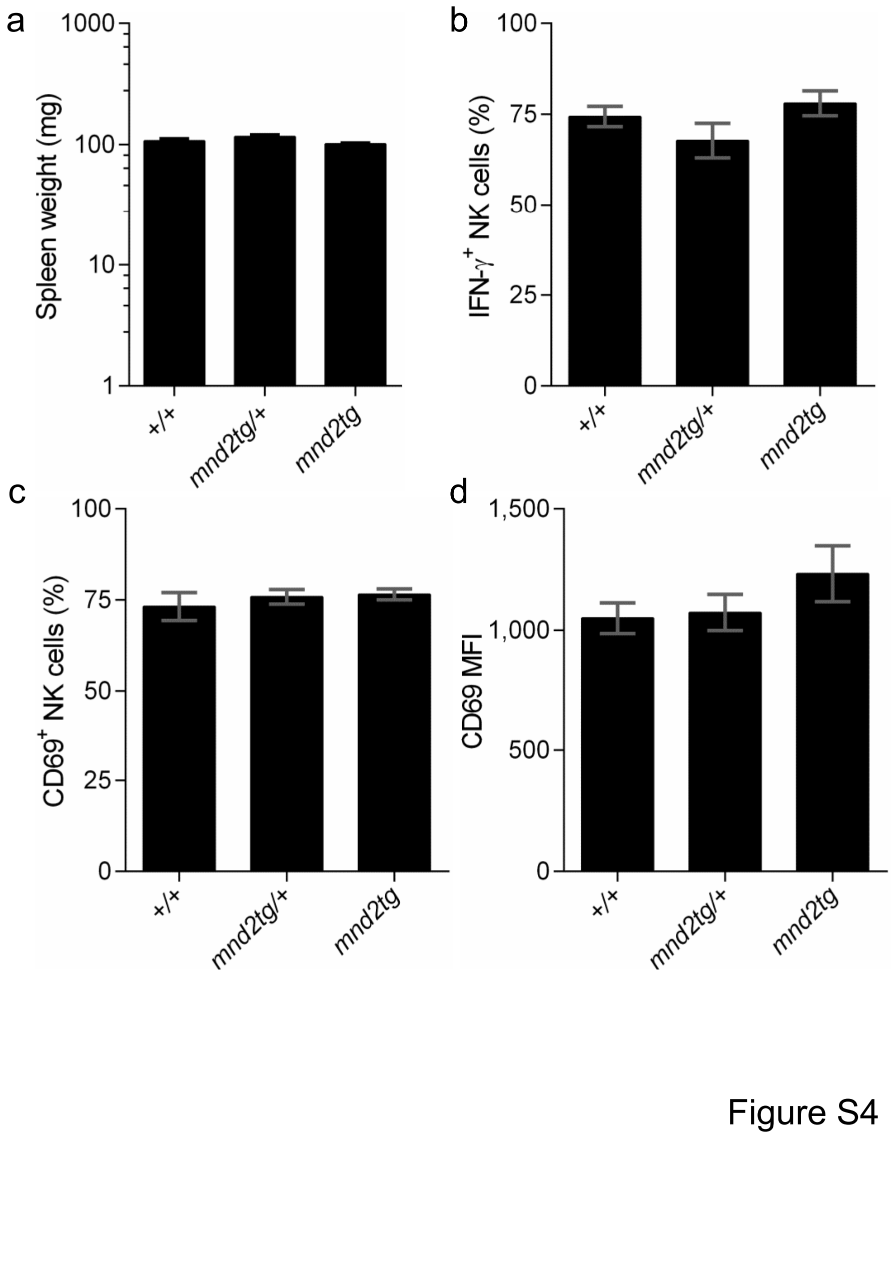
a
1000
100
10
1
Spleen weight (mg)
+/+
mnd2tg/+
mnd2tg
b
100
75
50
25
0
IFN-γ+ NK cells (%)
+/+
mnd2tg/+
mnd2tg
c
100
75
50
25
0
CD69+ NK cells (%)
+/+
mnd2tg/+
mnd2tg
d
1,500
1,000
500
0
CD69 MFI
+/+
mnd2tg/+
mnd2tg

Figure S4

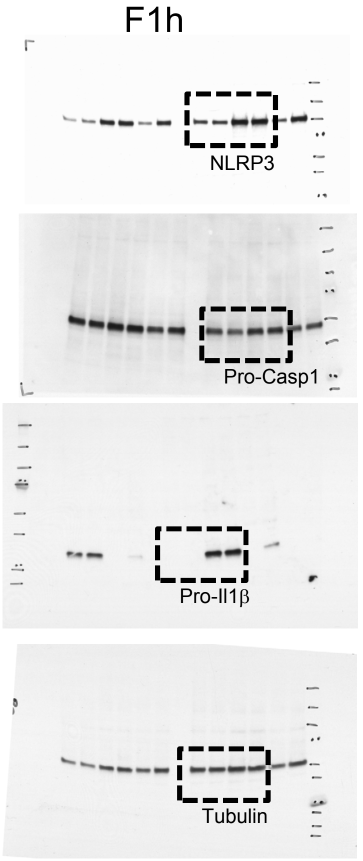

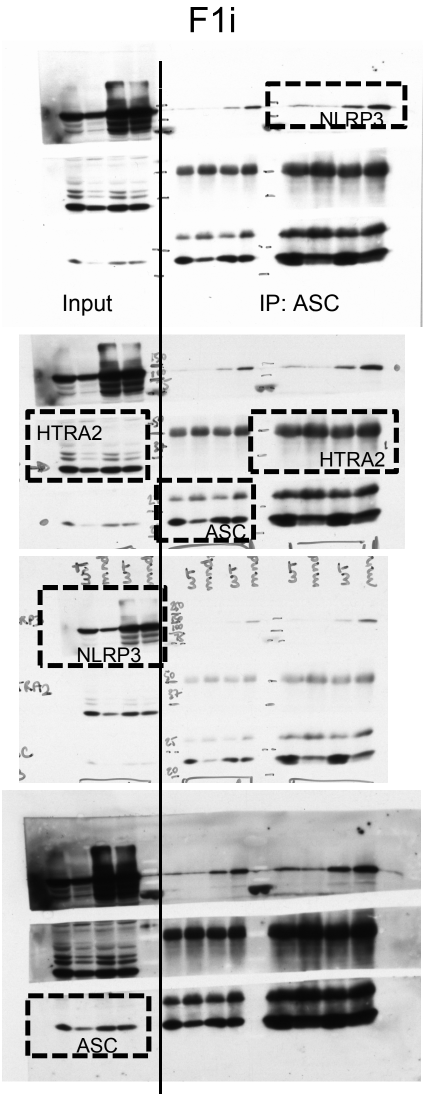

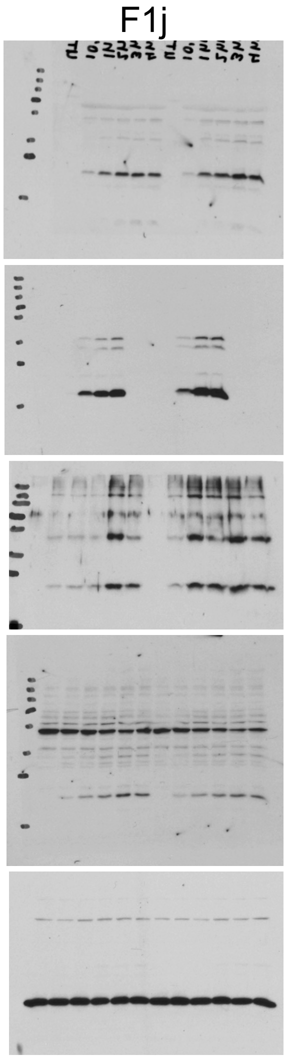

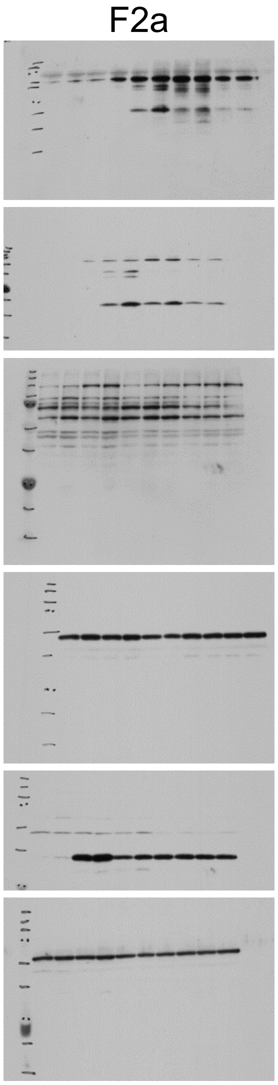

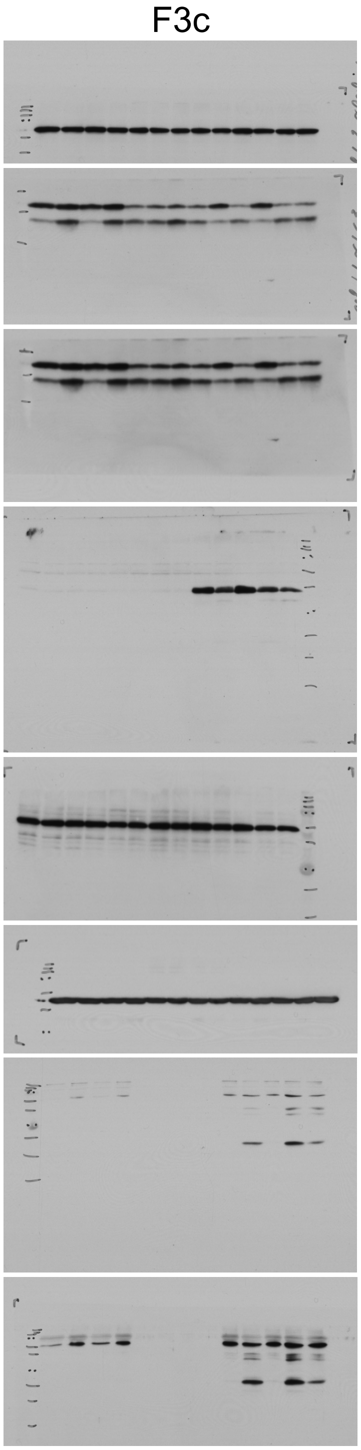

Figure Westernblots
